# Long-Term Use of Muscle Relaxant Medications for Chronic Pain: A Systematic Review

**DOI:** 10.1001/jamanetworkopen.2024.34835

**Published:** 2024-09-19

**Authors:** Benjamin J. Oldfield, Brynna Gleeson, Kenneth L. Morford, Zoe Adams, Melissa C. Funaro, William C. Becker, Jessica S. Merlin

**Affiliations:** 1 Program in Addiction Medicine, Department of Medicine, Yale School of Medicine, New Haven, Connecticut; 2 Fair Haven Community Health Care, New Haven, Connecticut; 3 Virginia Tech Carilion School of Medicine, Roanoke; 4 Department of Medicine, Massachusetts General Hospital, Boston; 5 Harvey Cushing/John Hay Whitney Medical Library, Yale University, New Haven, Connecticut; 6 Pain Research, Informatics, Multimorbidities, and Education (PRIME) Center, VA Connecticut Healthcare System, West Haven; 7 Challenges in Managing and Preventing Pain Clinical Research Center, University of Pittsburgh, Pittsburgh, Pennsylvania; 8 Division of General Internal Medicine, Center for Research on Health Care, University of Pittsburgh, Pittsburgh, Pennsylvania

## Abstract

**Importance:**

Stricter opioid prescribing guidelines have increased prescriptions of skeletal muscle relaxants (SMRs) for chronic pain, but the efficacy of long-term use of SMRs for chronic pain is unknown.

**Objective:**

To systematically review the effectiveness or efficacy of long-term use of SMRs for chronic pain.

**Evidence Review:**

Two reviewers systematically searched Ovid MEDLINE, Embase (Ovid), Web of Science, CINAHL, and Cochrane through December 4, 2023. They included articles published in English, Spanish, or Italian. Only randomized clinical trials (RCTs) and cohort studies with comparator groups evaluating at least 1-month duration of SMRs for chronic pain were included. The reviewers dually reviewed data abstraction, risk-of-bias, and quality. They characterized studies by chronic pain syndrome: low back pain, fibromyalgia, headaches, painful cramps or spasticity, and other syndromes.

**Findings:**

A total of 30 RCTs with 1314 participants and 14 cohort studies with 1168 participants assessed SMRs for chronic pain. Studies were primarily short-term (4-6 weeks). Nine unique SMRs were represented by the studies identified. Eleven studies (25%) examined baclofen, 8 (18%) examined tizanidine, and 7 (16%) examined cyclobenzaprine. Evidence for effectiveness was strongest for SMRs used for trigeminal neuralgia, neck pain, and painful cramps; evidence suggested SMRs for fibromyalgia, low back pain, and other syndromes were not more beneficial than placebo. The most common adverse effects were sedation and dry mouth. RCTs had a low to moderate risk of bias, and the quality of cohort studies was fair to good.

**Conclusions and Relevance:**

In this systematic review of long-term use of SMRs for chronic pain, findings suggest that their long-term use may benefit patients with painful spasms or cramps and neck pain; their long-term use for low back pain, fibromyalgia, and headaches did not appear to be beneficial. Clinicians should be vigilant for adverse effects and consider deprescribing if pain-related goals are not met.

## Key Points

**Question:**

What are the effectiveness and safety of long-term use of muscle relaxant medications for the treatment of chronic pain?

**Findings:**

In this systematic review of 44 studies including 2482 participants, 9 unique muscle relaxant medications were assessed. Muscle relaxants may be more beneficial than placebo for treating trigeminal neuralgia, painful cramps, and neck pain, but for fibromyalgia, low back pain, and other syndromes, they did not appear to be beneficial.

**Meaning:**

These findings suggest that long-term use of muscle relaxants may only be beneficial for certain syndromes; clinicians should consider deprescribing if pain-related goals are not met.

## Teaser

This systematic review evaluates the efficacy and safety of long-term use of skeletal muscle relaxants for low back pain, fibromyalgia, headaches, painful cramps or spasticity, and other syndromes.

## Introduction

Chronic pain, commonly defined as pain that lasts beyond 3 months and/or extends past normal tissue healing time,^1^ affects millions of US residents, with a 2021 prevalence of 21%.^2^ More than 50 million US adults experience pain most days or every day,^1^ making chronic pain one of the most significant public health problems in the United States.^3^ The Centers for Disease Control and Prevention’s *Clinical Practice Guideline for Prescribing Opioids for Pain*^4,5^ and the Department of Health and Human Services’ *National Pain Strategy*^6^ call for a multimodal approach to pain management, incorporating nonpharmacologic and nonopioid pharmacologic treatment options, leaving considerable latitude for shared decision-making between patients and clinicians. Guidelines specific to certain pain syndromes, such as the American College of Physicians’ clinical practice guideline on treatments for low back pain, emphasize nonopioid medications including nonsteroidal anti-inflammatories (NSAIDs) and muscle relaxant medications.^7^

Centrally acting skeletal muscle relaxants (SMRs) are a pharmacologically diverse category of medications that include antispasticity and antispasmodic medications, such as baclofen, carisoprodol, chlorzoxazone, cyclobenzaprine, metaxalone, methocarbamol, orphenadrine, and tizanidine. They are indicated for acute musculoskeletal conditions including spasms and low back pain; they are also used off-label for numerous other pain and nonpain conditions.^8^ SMRs are to be used with caution because of central nervous system adverse effects, including drowsiness and dizziness, particularly when used in combination with other centrally acting medications.^9^ Because of these adverse effects and a lack of evidence regarding the long-term efficacy of SMRs, recommendations generally limit their use to a maximum duration of 2 to 3 weeks.^9^ However, SMR prescribing doubled between 2005 and 2016, and physician visits for continuing SMR prescriptions tripled during the same period, indicating a shift toward longer duration of use and for nonacute (including chronic) pain syndromes.^10^

Prior systematic reviews^9,11^ on the effectiveness or efficacy of longer-term use of SMRs were conducted before this growth of use, focused on specific conditions such as low back pain, and were limited to clinical trials. Given the increase in use of this medication class, and because approximately one-third of patients being prescribed SMRs do not have a preceding musculoskeletal disorder diagnosis,^12^ a broader examination of their long-term use for multiple chronic conditions is needed. The aim of this systematic review was to evaluate the effectiveness or efficacy of long-term (≥4 weeks) use of SMRs for chronic (≥3 months) pain.

## Methods

The reporting of this systematic review was guided by the Preferred Reporting Items for Systematic Reviews and Meta-Analyses (PRISMA) standards of quality.^13^ We developed a protocol for study eligibility a priori and registered it in the PROSPERO database of systematic reviews (CRD42019128973).^14^ This study was not considered human participant research by the Yale School of Medicine Human Investigation Committee.

### Analysis Team

We crafted a research team to draw from multiple sources of expertise.^15^ Our team included clinician-investigators who provide primary care (B.J.O. and K.L.M.), specialized addiction treatment (B.J.O., K.L.M., J.S.M., and W.C.B.), specialized pain management (W.C.B. and J.S.M.), and specialized palliative care (J.S.M.) as well as a medical librarian with experience in systematic reviews (M.C.F.) and medical trainees (B.G. and Z.A.).

### Data Sources and Searches

Our search was structured around the following domains: (1) population of interest (painful syndrome lasting ≥3 months); (2) intervention of interest (use of a nonbenzodiazepine SMR for ≥4 weeks); (3) an adequate comparator; (4) outcomes pertaining to pain severity, pain interference, or quality of life; and (5) study type (randomized clinical trial or cohort study involving at least 10 participants). These inclusion criteria are summarized in Table 1.

**Table 1.** Inclusion Criteria Following the Population, Intervention, Comparison, Outcome, and Study Design Framework

Element	Inclusion criteria
Population	Adults ages ≥18 y with chronic pain or painful muscle spasms (experiencing pain on most days for >3 mo)
Intervention	Daily use of an oral nonbenzodiazepine antispasmotic drug(s) for 4 weeks or longer
Comparison	Placebo, other pain medication, nonpharmacologic pain treatment, nonexposed cohort, baseline (historical) data
Outcome	Any outcome(s) that pertains to pain severity, pain interference, or quality of life
Study design	Randomized clinical trials, observational studies with a nonexposed cohort, observational studies with baseline evaluation, with at least 10 patients in the intervention arm

We performed a comprehensive search of the following databases: Ovid MEDLINE, Embase (Ovid), Web of Science, CINAHL, and Cochrane. We performed all searches on December 4, 2023. Search results were pooled in EndNote version 21 (Clarivate) and duplicates removed before uploading to Covidence, a systematic review software. We identified additional studies by scanning other systematic reviews and bibliographies. We also searched the websites of the following preidentified organizations for appropriate references to studies that may not have been indexed in the databases above (ie, grey literature): Society for General Internal Medicine, Substance Abuse and Mental Health Services Administration, and the American Academy of Pain Medicine. We limited our search to studies with human participants and those published in English, Spanish, or Italian. We did not impose a date-of-publication restriction on study inclusion.

To produce relevant controlled vocabulary and keyword terms, we analyzed 5 previously identified key articles using the Yale MeSH Analyzer.^16^ In each database, we ran scoping searches and used an iterative process to translate and refine the search strategies. We used the previously identified articles to validate the success of our searches (for exact search terminology and syntax, see the eAppendix in Supplement 1).

### Study Selection

Two authors independently screened titles and abstracts using a screening algorithm developed a priori. All disagreements were resolved by consensus with the input from the first author. We used Covidence, a systematic review software, to facilitate screeners’ independent organization, retrieval, and assessment of articles.^17^

### Data Extraction and Quality Assessment

For each screened article, 2 authors independently abstracted information about the context, participants, intervention, and outcomes into a standardized form. If desired information was not published, we contacted the first author of the article by email to inquire. To pool the varied expertise on our team, at least 2 team members read all screened studies. As we anticipated considerable heterogeneity of settings in which included studies may have taken place, our data synthesis process drew from realist synthesis, an analytic approach driven by realist theory that considers the interaction between context, mechanism, and outcome in evaluating an intervention.^18,19,20^ In the realist synthesis strategy, reviewers delineated the contextual influences (C) that were hypothesized to have contributed to the relevant mechanisms (R) to generate the outcomes (O) of interest.^19,20^ Contextual influences, in this case, were defined by the type of pain syndrome identified. We arrived at consensus for C-R-O sequences during iterative consultations among members of the research team.

Two reviewers independently completed the quality assessment of each study using the Cochrane Risk of Bias Tool for randomized clinical trials^21^ and the Newcastle-Ottawa Scales for observational studies.^22^ Disagreements were resolved by consensus with the input of the first author.

### Data Analysis

Like previous systematic reviews of the effectiveness of interventions on chronic pain conditions^9,11,23^ and further informed by the variety of studies identified, we classified interventions by pain syndrome groupings. The groupings were low back pain, fibromyalgia, headaches, painful cramps or spasticity, and other syndromes.

## Results

Our search yielded 21 889 articles, and 14 128 remained after the removal of duplicates. Following title and abstract screens, we screened 177 full-text articles for eligibility and identified 44 articles that met criteria for inclusion, each representing a unique study (Figure).^24,25,26,27,28,29,30,31,32,33,34,35,36,37,38,39,40,41,42,43,44,45,46,47,48,49,50,51,52,53,54,55,56,57,58,59,60,61,62,63,64,65,66,67^ These included 30 randomized clinical trials^24,25,26,27,28,29,30,31,32,33,34,35,36,37,39,41,45,46,48,50,51,53,54,58,59,60,61,64,65,67^ with 1314 participants and 14 cohort studies^38,40,42,43,44,47,49,55,56,57,62,63,66^ with 1168 participants.

**Figure.**
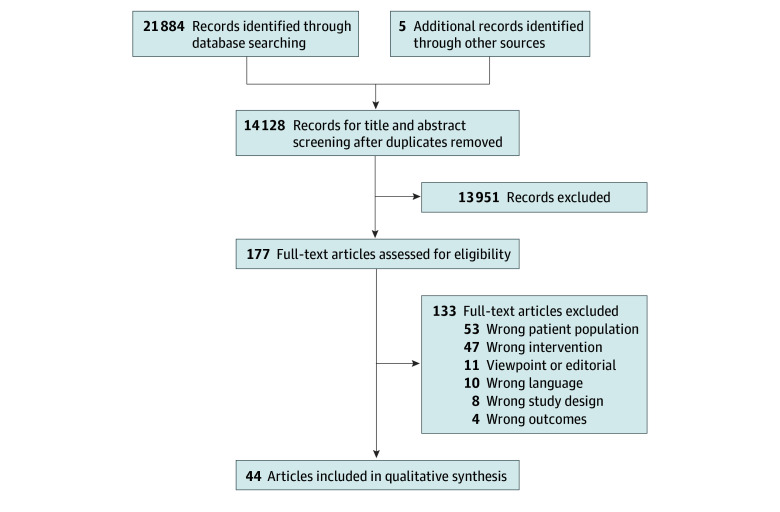
Flowchart of Study Selection

### Description of Studies

We identified 5 studies that addressed low back pain (eTable 1 in Supplement 1),^53,54,60,64,66^ 11 studies that addressed fibromyalgia or related disorders (eTable 2 in Supplement 1),^28,31,32,41,42,43,48,51,52,59,62^ 10 studies that addressed headaches or trigeminal neuralgia (eTable 3 in Supplement 1),^27,37,40,45,46,47,50,56,57,67^ 10 studies that addressed painful muscle cramps or spasticity (eTable 4 in Supplement 1),^24,25,26,34,36,38,49,58,61^ and 8 studies that addressed other pain syndromes, including osteoarthritis,^29,39^ cervical spondylosis,^30^ neuropathy,^35,55^ cancer pain,^63^ gastric reflux–related pain,^65^ and orchialgia^44^ (eTable 5 in Supplement 1).

Nine muscle relaxant medications were represented by the studies identified. The most common were as follows: 11 studies (25%) examined baclofen,^26,36,38,44,47,50,57,63,64,65,67^ 8 (18%) examined tizanidine,^37,40,42,43,46,55,56,62^ and 7 (16%) examined cyclobenzaprine.^28,31,32,35,41,45,51^ Other studies examined eperisone,^30,39,52,53,54^ quinine,^34,60,61^ carisoprodol,^33,58,59^ orphenadrine,^25,27,49^ chlormezanone,^29,48^ and methocarbamol.^24,66^

While a plurality of studies took place in the United States or Canada, 12 took place in Europe,^29,31,37,46,48,52,53,57,59,60,65,66^ 12 took place in Asia,^26,30,35,39,44,47,50,54,56,63,64,67^ 3 in Africa,^24,25,36^ and 1 in Australia/New Zealand.^61^ Other characteristics of the included studies are found in Table 2.

**Table 2.** Characteristics of 44 Included Studies

Characteristic	Studies, No. (%) (N = 44)
Study type	
Randomized clinical trial	30 (68)
Cohort study	14 (32)
Study region	
United States or Canada	16 (36)
Europe	12 (27)
Asia	12 (27)
Africa	3 (6)
Australia/New Zealand	1 (2)
Year of publication	
2020-2023	4 (9)
2010-2019	11 (25)
2000-2009	13 (30)
1990-1999	6 (14)
1980-1989	7 (16)
1970-1979	1 (2)
1960-1969	2 (4)
Pain syndrome	
Low back pain	5 (11)
Fibromyalgia	11 (25)
Headache or trigeminal neuralgia	10 (23)
Cramps or painful spasticity	10 (23)
Other	8 (18)
Muscle relaxant	
Baclofen	11 (25)
Tizanidine	8 (18)
Cyclobenzaprine	7 (16)
Eperisone	5 (11)
Quinine	3 (7)
Carisoprodol	3 (7)
Orphenadrine	3 (7)
Chlormezanone	2 (4)
Methocarbamol	2 (4)
Intervention duration	
4 wk	13 (30)
4-12 wk	24 (56)
>12 wk	7 (16)

### Quality Assessment

The risk of bias among randomized clinical studies was low to moderate (eTable 6 in Supplement 1). Risk of bias most commonly manifested as lack of blinding of participants and personnel as well as lack of blinding of outcomes assessments. Cohort studies were of fair to good quality (eTable 7 in Supplement 1). The most common reasons for low quality assessment were low comparability of cohorts based on design or analysis (13 of 14) and low-quality selection of the nonexposed cohort (12 of 14).

### Interventions for Back Pain

Among the 5 studies of interventions for back pain, 4 were RCTs^53,54,60,64^ and included a total of 98 patients in the intervention arms; 1 cohort study^66^ included 374 individuals in the intervention arm (eTable 1 in Supplement 1). Two of the 5 studies involved eperisone,^53,54^ 1 involved baclofen,^64^ 1 involved quinine,^60^ and 1 involved methocarbamol.^66^ Eperisone with tramadol was not associated with improvements in pain severity more than tizanidine with tramadol.^53^ In a study comparing eperisone with physical therapy and McKenzie therapy (a form of physical therapy that approaches the type of symptomatic complaint more than the anatomic location of the pain), pain scores were most improved in the McKenzie group.^54^ When quinine was compared with placebo for back pain related to ankylosing spondylitis, mean scores for pain and function did not differ between groups.^60^ In a study comparing baclofen with placebo and with baclofen and acupuncture, pain scores decreased in all groups but returned to baseline in the baclofen-only group 5 weeks after discontinuation, whereas scores remained improved in the baclofen with acupuncture group.^64^ In a propensity score–matched (cohort) study comparing methocarbamol with long-term oral opioid analgesics,^66^ both arms showed clinical improvement, with superior improvement and fewer adverse events in the methocarbamol group. Prevalence of adverse effects in eperisone groups ranged from none to 17% and included somnolence. Adverse effects in the methocarbamol group occurred in 10% of patients and included somnolence and dizziness. No adverse effects were documented for quinine nor baclofen.

### Interventions for Fibromyalgia and Similar Disorders

Among 11 studies of intervention for fibromyalgia and similar disorders, 7 were RCTs^28,31,32,41,48,51,59^ and 4 were cohort studies^42,43,52,62^ and included a total of 391 patients (eTable 2 in Supplement 1). Five studies involved cyclobenzaprine,^28,31,32,41,51^ 3 involved tizanidine,^42,43,62^ and 1 study each involved chlormezanone,^48^ eperisone,^52^ and carisoprodol.^59^ Among those involving cyclobenzaprine, all were RCTs. In 3 studies,^28,41,51^ cyclobenzaprine was associated with improvement in sleep disturbance but with no difference from placebo in other outcomes. In an RCT comparing cyclobenzaprine with amitriptyline,^32^ both groups improved clinically over 6 months with no difference between groups. Prevalence of adverse effects ranged from none to 98% and included somnolence, dry mouth, and, for those taking a sublingual formulation, tongue and sublingual numbness. Among studies examining tizanidine for fibromyalgia,^42,43,62^ all were cohort studies, and documented improvements in pain intensity for participants beyond baseline; 1 study^43^ examined outcomes 1 week after tizanidine was stopped and noted that pain intensity worsened again. Prevalence of adverse effects were none to 66% and included somnolence, headaches, and dizziness. In RCTs examining chlormezanone and carisoprodol, pain was not improved in the intervention groups compared with placebo. A cohort study involving eperisone^52^ showed improvements in pain scales compared with celecoxib at 2, 4, and 6 weeks of treatment. Chlormezanone was associated with nausea (prevalence 48%); no adverse effects were noted for carisoprodol or eperisone.

### Interventions for Headaches

Among 10 studies of interventions for headaches, including trigeminal neuralgia, 6 were RCTs^27,37,45,46,50,67^ and 4 were cohort studies,^40,47,56,57^ including a total of 558 patients in the intervention arms (eTable 3 in the Supplement). Four studies focused on tizanidine,^37,40,46,56^ 3 on baclofen,^47,50,57^ and 1 each on cyclobenzaprine^45^ and orphenadrine.^27^ Studies involving tizanidine demonstrated improvement from baseline in pain severity; 1 six-week RCT^37^ demonstrated improvement vs placebo, and another, also a 6-week RCT,^46^ did not. Drowsiness, dry mouth, vivid dreams, and hallucinations were reported in tizanidine groups; in 1 study,^40^ 25% of participants in the tizanidine group dropped out due to adverse effects. Studies involving baclofen demonstrated improvement from baseline; 1 study^50^ comparing baclofen and carbamazepine vs carbamazepine alone found improved reduction of pain in the combination group. Adverse effect incidence ranged from none to 35% and included sedation, vomiting, diarrhea, nausea, weakness, and constipation. Orphenadrine compared with diazepam^27^ and cyclobenzaprine compared with placebo^45^ did not confer improved reductions in symptoms.

### Interventions for Painful Cramps or Spasticity

Among 10 studies of interventions for painful cramps or spasticity, 8 were RCTs^24,25,26,34,36,58,61^ and 2 were cohort studies^38,49^; they included 330 patients in total in the intervention arms (eTable 4 in Supplement 1). Six addressed nocturnal leg cramps,^24,25,34,36,38,49^ with 4 of these^24,25,36,38^ among patients with cirrhosis of the liver. Overall, 3 focused on baclofen,^26,36,38^ 2 focused on orphenadrine,^25,49^ 2 on carisoprodol,^33,58^ 2 on quinine,^34,61^ and 1 on methocarbamol.^24^ Baclofen was associated with significant improvements in cramp frequency, duration, and severity compared with placebo, but not when compared with transcutaneous electrical nerve stimulation. Orphenadrine, carisoprodol, and methocarbamol were associated with improved cramp frequency beyond placebo. Adverse effects were documented for the baclofen, orphenadrine, and carisoprodol groups.

### Interventions for Other Syndromes

The 8 studies of intervention for other syndromes addressed osteoarthritis, cervical pain, orchialgia, pain associated with cancer, neuropathic pain, gastric reflux–related pain, and osteroarthritis of multiple joints (eTable 5 in Supplement 1). Chlormezanone was associated with reduced number of breaks in sleep among those with neck osteoarthritis, but not osteoarthritis of the hip, knee, lumbar spine, or shoulder.^29^ Eperisone was also associated with improved neck pain over placebo at 6 weeks.^30^

## Discussion

This systematic review identified 44 studies that investigated the long-term use of SMRs, including 9 specific medications, for a range of chronic pain conditions. Evidence for effectiveness was strongest for SMRs used for muscle spasms, painful cramps, and neck pain; in studies of SMRs for fibromyalgia, low back pain, headaches, and other syndromes, some showed small benefits and some did not, and on balance studies did not suggest a benefit. The most common adverse effects were sedation (and other central nervous system–related effects, including dizziness) and dry mouth. No studies measured the misuse of SMRs. Most studies lasted only a month or slightly longer, and this short duration may bias toward higher efficacy (many pharmacologic treatments for pain show declining efficacy over time) and toward lower adverse effects (which may develop over time, including misuse of SMRs).

This summary of the evidence raises concerns given the growth in SMR prescriptions over the last decade,^10^ including for more than 1 in 6 patients seeking care for chronic back pain in a national study of Medicare beneficiaries.^68^ Furthermore, previous studies suggest that as many as 30% of individuals using opioids are also prescribed SMRs,^69,70^ which increase risks of opioid-related overdose, particularly in those taking SMRs for longer durations.^71^ Therefore, despite increasing prevalence and increasing risks of their use, our systematic review suggests only limited evidence of efficacy for long-term use of SMRs for a small subset of pain syndromes.

This review broadens the work of prior reviews that have focused on individual medications and specific pain syndromes. Two recent systematic reviews^23,72^ examining the effectiveness and safety of multiple medication classes in the management of acute and chronic nonspecific lower back pain found no evidence of difference between SMRs and placebo in the management of chronic nonspecific low-back pain. Another review^73^ focused on the efficacy and safety of cyclobenzaprine for myofascial pain and found insufficient evidence to support the use of cyclobenzaprine. While nonspecific back pain tends to be the most common reason for SMR prescriptions in the United States, approximately one-third of office visits during which SMRs were prescribed addressed non–back pain syndromes, suggesting that in practice SMRs are used for a variety of indications for which evidence is limited.^10^

Most studies included in this review examined the efficacy of SMRs in comparison with placebo or, in the case of cohort studies, in comparison with a historical control. However, the painful syndromes studied in this review have effective therapies available, against whose efficacy SMRs should be measured. For example, in the case of chronic nonspecific low-back pain, moderate-quality evidence supports the effectiveness of exercise, multidisciplinary rehabilitation, acupuncture, and mindfulness-based stress reduction.^7^ In the case of fibromyalgia, graded exercise is the mainstay of therapy, and among pharmacotherapy, tricyclic antidepressants and serotonin-norepinephrine reuptake inhibitors have meta-analytic evidence of efficacy.^74,75,76^ For patients already prescribed long-term SMRs, interventions are needed to assist clinicians to engage in shared decision-making with patients about deprescribing SMRs. This may be particularly true for older patients, for whom risks of adverse events may be greater. Academic detailing and tapering guidelines, which have shown some success in deprescribing of opioids and other medications, may inform these interventions.^77,78^

### Limitations

This systematic review was limited to only English-, Spanish-, and Italian-language publications, so studies from countries where these languages are not spoken, including low- and middle-income countries, may not have been included. The varying nature of the clinical sites, pain syndrome definitions, qualifying medications, and durations of therapy precluded meta-analyses, so we used a narrative synthesis based on a realist framework to identify the evidence for different clinical contexts.^20^ Finally, because we included only quantitative studies to facilitate comparison across studies, we did not include qualitative studies that may offer valuable insights into complex care processes and patient experiences,^79^ which are particularly important in pain management.^80^

## Conclusions

This systematic review identified 44 studies examining the effectiveness or efficacy of long-term use of SMRs to treat chronic painful conditions, a clinical practice that has expanded considerably in recent decades. Long-term use of SMRs for chronic pain may be beneficial for patients with painful spasms or cramps and neck pain; evidence was equivocal for their long-term use for low back pain, fibromyalgia, and headaches. Clinicians should be vigilant for adverse effects and consider deprescribing if pain-related goals are not met.

## Notes

Supplement 1. **eAppendix**. Embase Search Strategy Syntax **eTable 1. **Characteristics of Interventions for Low Back Pain **eTable 2. **Characteristics of Interventions for Fibromyalgia and Similar Disorders **eTable 3. **Characteristics of Interventions for Headaches or Trigeminal Neuralgia **eTable 4. **Characteristics of Interventions for Painful Muscle Cramps or Spasticity **eTable 5. **Characteristics of Interventions for Other Pain Syndromes **eTable 6**. Risk of Bias Assessment for Randomized Trials (Cochrane Risk of Bias Tool) **eTable 7**. Quality Assessment for Cohort Studies (Newcastle-Ottawa Scale)

Supplement 2.
Data Sharing Statement
