# Supplementary material for: Long-Term Use of Muscle Relaxant Medications for Chronic Pain: A Systematic Review
Source: JAMA Netw Open. 2024 Sep 19;7(9):e2434835. doi: 10.1001/jamanetworkopen.2024.34835 (PMC11413720; doi:10.1001/jamanetworkopen.2024.34835)
Supplement: Supplement 1. — eAppendix. Embase Search Strategy Syntax eTable 1. Characteristics of Interventions for Low Back Pain eTable 2. Characteristics of Interventions for Fibromyalgia and Similar Disorders eTable 3. Characteristics of Interventions for Headaches or Trigeminal Neuralgia eTable 4. Characteristics of Interventions for Painful Muscle Cramps or Spasticity eTable 5. Characteristics of Interventions for Other Pain Syndromes eTable 6. Risk of Bias Assessment for Randomized Trials (Cochrane Risk of Bias Tool) eTable 7. Quality Assessment for Cohort Studies (Newcastle-Ottawa Scale) [file jamanetwopen-e2434835-s001.pdf]

Supplemental Online Content

Oldfield BJ, Gleeson B, Morford KL, et al. Long-term use of muscle relaxant medications for chronic pain. *JAMA Netw Open.* 2024;7(9):e2434835. doi:10.1001/jamanetworkopen.2024.34835

eAppendix. Embase Search Strategy Syntax

eTable 1. Characteristics of Interventions for Low Back Pain

eTable 2. Characteristics of Interventions for Fibromyalgia and Similar Disorders

eTable 3. Characteristics of Interventions for Headaches or Trigeminal Neuralgia

eTable 4. Characteristics of Interventions for Painful Muscle Cramps or Spasticity

eTable 5. Characteristics of Interventions for Other Pain Syndromes

eTable 6. Risk of Bias Assessment for Randomized Trials (Cochrane Risk of Bias Tool)

eTable 7. Quality Assessment for Cohort Studies (Newcastle-Ottawa Scale)

This supplemental material has been provided by the authors to give readers additional information about their work.

© 2024 Oldfield BJ et al. *JAMA Network Open.*

eAppendix. Embase Search Strategy Syntax

1	muscle relaxants, central/
2	(muscle relax* or anti spasm* or calmative or afloqualone or arbaclofen placarbil or baclofen or baclophen or bendazole or carisoprodol or chlormezanone or chlorphenesin or chlorproethazine or chlorzoxazone or cyclobenzaprine or dantrium or dantrolene or dibazole or disipal or eperisone or flexeril or gyki 52466 or lioresal or mephenesin or metaxalone or methocarbamol or mydeton or mydocalm or norflex or norflex or norgesic or orphenadrine or parafon or quinamm or quine or quinine or rilmakalim or robaxin or rompun or tizanidine or tolperisone or xylaxine or xylazin or xylazine or y 27632 or zanaflex or zoxazolamine).tw,kw.
3	exp pain/
4	(pain or pains or painful or arthralgia or back dysfunction or failed back surgery syndrome or lumbago or backache* or headache or head ache or migraine* or mastalgia* or mastodynia* or mammalgia* or metatarsalgia or myalgia or neck ache or neckache or neuralgia or neuralgias pudendal nerve entrapment or sciatica or dysmenorrhea or painful menstruation* or symphysis pubis dysfunction or (back adj (strain* or syndrome* or ache*)) or (Morton* adj (disease or neuroma or neuralgia)) or (piriformis adj2 syndrome)).tw,kw.
5	exp "sprains and strains"/ or exp muscle cramp/ or exp muscle hypertonia/ or exp myotonia/ or exp spasm/ or exp tetany/
6	(sprain or sprains or strain or strains or cumulative trauma disorder* or (repetitive adj injur*) or overuse injur* or carpal tunnel or (iliotibial adj2 syndrome) or nerve compression or ulnar neuropath* or (muscle adj (cramp* or hypertonia or rigidity or spasticity)) or (muscular adj (cramp* or hypertonia or rigidity)) or cogwheel rigidity or myotonia or myotonias or spasm or spasms or lock jaw or lockjaw or trismus or tetany).tw,kw.
7	1 or 2
8	3 or 4 or 5 or 6
9	7 and 8
10	9 not (exp animals/ not humans.sh.)

© 2024 Oldfield BJ et al. *JAMA Network Open.*

eTable 1. Characteristics of Interventions for Low Back Pain

Medication	First author, country, publication year	Study design	Intervention description	Number of patients in intervention	Key findings
Eperisone	Rossi<br>Italy<br>2012	RCT	Patients with low-back pain randomized to eperisone with tramadol vs. tizanidine with tramadol for 30 days	30 received eperisone with tramadol; 30 received tizanidine with tramadol)	- Significant reduction in visual analog pain scales noted at five, 10, 15, and 30 days in both groups.<br>- No significant differences were noted in the improvement reported between groups.<br>- Somnolence noted more frequently in tizanidine with tramadol group (13, 43%) compared to eperisone with tramadol (5, 17%).<br>- Treatment discontinued in 5 (17%) patients in eperisone with tramadol group and in 9 (30%) of tizanidine with tramadol group due to adverse effects.
	Sakai<br>Japan<br>2008	RCT	Patients low-back pain randomized to eperisone vs. physical therapy vs. McKenzie therapy for four weeks	24<br>(25 received physical therapy, 25 received McKenzie therapy	- Significant increase in intramuscular oxygenation noted in the eperisone group at four weeks.<br>- Pain scores significantly improved in McKenzie group compared to physical therapy and eperisone group.<br>- No adverse effects reported in any group
Quinine	Williamson<br>UK<br>2000	Crossover RCT	Patients with painful ankylosing spondylitis randomized to quinine vs. placebo for four week periods	24	- Mean scores for all outcomes measures (disease index, functional index, and metrology index) not significantly different during quinine periods.<br>- No adverse effects noted during quinine or placebo periods.
Baclofen	Zaringhalam<br>Iran<br>2010	RCT	Patients with low-back pain randomized to control, baclofen, acupuncture, or baclofen with	20<br>(20 in control, 20 in baclofen group, and 20 in baclofen with acupuncture group)	- Visual analog pain scores decreased significantly in all treatment groups.<br>- Pain scores returned to baseline in baclofen group after discontinuation; scores remained significantly improved in acupuncture and acupuncture with baclofen groups five weeks after study conclusion.<br>- Adverse effects were not commented on.

© 2024 Oldfield BJ et al. *JAMA Network Open.*

			acupuncture for five weeks		
Methocarbamol	Ueberall Germany 2022	Cohort	Propensity score-matched data analyzed for patients with non-specific low-back pain treated with methocarbamol versus long-acting oral opioid analgesics for four weeks	374 (374 received long-acting oral opioid analgesics)	- Visual analog pain scores decreased significantly in both groups; improvement vs baseline was superior for methocarbamol versus long-acting oral opioids (effect size 1.6).<br>- Significantly lower number of patients on methocarbamol versus long-acting oral opioid analgesics reported drug-related adverse events: 36 (9.6%) versus 139 (37.2%).

© 2024 Oldfield BJ et al. *JAMA Network Open.*

eTable 2. Characteristics of Interventions for Fibromyalgia and Similar Disorders

Medication	First author, country, publication year	Study design	Intervention description	Number of patients in intervention	Key findings
Cyclobenzaprine	Bennett<br>USA<br>1988	RCT	Patients with fibrositis (sic) randomized to cyclobenzaprine vs. placebo for 12 weeks	62<br>(58 received placebo)	- Patients in cyclobenzaprine group significantly more likely to report improvement in pain severity and sleep disturbance.<br>- No significant differences between groups regarding fatigability, morning stiffness.<br>- Among those in placebo group, 52% withdrew due to lack of beneficial response, compared to 16% those in cyclobenzaprine group.
	Cantini<br>Italy<br>1993	RCT	Female patients with fibromyalgia randomized to cyclobenzaprine with or without fluoxetine for 12 weeks	21	- Significant improvement at 12 weeks (compared to baseline) in pain scores, tender points, and morning stiffness in both groups.<br>- Degree of improvement significantly greater among cyclobenzaprine with fluoxetine in pain scores, tender points, and morning stiffness.<br>- Adverse effects not commented on.
	Carette<br>Canada<br>1994	RCT	Patients with fibromyalgia randomized to amitriptyline, cyclobenzaprine, or placebo for six months	82<br>(82 received amitriptyline, 42 received placebo)	- At one month, significant clinical improvement in both amitriptyline and cyclobenzaprine groups over placebo; no different between amitriptyline and cyclobenzaprine.<br>- At six months, improvement noted in all groups with no significant differences.<br>- Adverse effects reported in 95% of the amitriptyline group, 98% of the cyclobenzaprine group, and 62% of the placebo group.
	Lederman<br>USA<br>2015	RCT	Patients with fibromyalgia randomized to rapidly absorbed, sublingual cyclobenzaprine vs. placebo for 12 weeks	103<br>(102 received placebo)	- Pain response (defined as ≥30% improvement from baseline) reported in 34% of cyclobenzaprine group vs. 21% placebo group ($P = 0.03$).<br>- Multiple measures of sleep quality significantly better in cyclobenzaprine group vs. placebo.<br>- Systemic adverse events not reported, but 42% of those in cyclobenzaprine group reported local reactions (tongue or sublingual numbness).

© 2024 Oldfield BJ et al. *JAMA Network Open.*

	Quimby<br>USA<br>1989	RCT	Female patients with fibromyalgia randomized to carbamazepine vs. placebo for six weeks	23<br>(20 received placebo)	- Significantly better improvement in stiffness, sleep and overall patient & physician rating reported in the cyclobenzaprine group.<br>- No significant differences in improvement in fatigue and pain scores between groups.<br>- No patients dropped out; dry mouth reported in 6 (26%) of those in cyclobenzaprine group.
Chlormezanone	Pattrick<br>UK<br>1993	RCT	Patients with fibromyalgia randomized to chlormezanone vs. placebo for six weeks	21<br>(21 received placebo)	- No benefit was seen in chlormezanone vs. placebo in total tender sites, alertness, pain, sleep, morning stiffness, fatigue, and global opinion.<br>- Seven (33%) patients in placebo group and 10 (48%) in chlormezanone group reported adverse effects; nausea most common in chlormezanone group.
Eperisone	Reale<br>Italy<br>2012	Cohort	Patients with fibromyalgia given eperisone or celecoxib for six weeks	15<br>(15 received celecoxib)	- Significant improvement in numerical rating scale noted in both groups.<br>- Lower scale results noted in eperisone group compared to celecoxib group at two, four, and six weeks of treatment.<br>- No adverse effects observed.
Tizanidine	Malanga<br>USA<br>2002	Cohort	Female patients with myofascial pain syndrome given tizanidine for five weeks	29	- Mean pain questionnaire score significantly improved, compared to baseline, at week 3, 5, and endpoint.<br>- Mean pain intensity score significantly improved, compared to baseline, at week 3, 5, and endpoint.<br>- All scores worsened one week after completion.<br>- Overall, 19 (66%) experienced adverse events: somnolence (28%), headache (24%), and dizziness (24%).
	McLain<br>USA<br>2002	Cohort	Patients with fibromyalgia given tizanidine for 14 weeks	46	- At seven weeks, significant improvement in tender points, visual analog pain scale and visual analog fatigue scale.<br>- At 14 weeks, improvement limited to tender points and fatigue scale.<br>- Among those who were disabled or applying for disability, no improvement noted.<br>- Among 46, only 30 (65%) completed the study. Eight (17%) dropped out due to adverse events, not commented upon.
	Xiao<br>2002<br>USA	Cohort	Patients with fibromyalgia given tizanidine for eight weeks	25	- Cerebrospinal fluid substance P levels decreased significantly during the study period.<br>- Significant improvements noted in sleep, depression, and pain mentioned but not quantified
Carisoprodol	Vaerøy<br>Norway<br>1988	RCT	Patients with fibromyalgia randomized to	20<br>(23 received placebo)	- Improvement noted in both groups in pain and sleep visual analog scores. Improvement noted in "general feeling of sickness" scores in drug but not placebo group.

© 2024 Oldfield BJ et al. *JAMA Network Open.*

			carisoprodol, acetaminophen and caffeine vs. placebo for eight weeks		- No significant differences in pain, sleep or general feeling of sickness between the two groups.<br>- No serious adverse effects reported.

© 2024 Oldfield BJ et al. *JAMA Network Open.*

eTable 3. Characteristics of Interventions for Headaches or Trigeminal Neuralgia

Medication	First author, country, publication year	Study design	Intervention description	Number of patients in intervention	Key findings
Orphenadrine	Bakris<br>USA<br>1982	RCT	Women with newly diagnosed chronic muscle-contraction headaches randomized to orphenadrine vs. diazepam for six months	19<br>(19 received diazepam)	- Eighteen (95%) patients in the orphenadrine group reported improvement at one week, 16 (84%) at one month, and 14 (74%) at six months. In the diazepam group, results were 17 (90%), 15 (79%), and 15 (79%), respectively.<br>- While drowsiness, weight gain, and rash were reported in the diazepam group, none of these were reported in the orphenadrine group.<br>- Nineteen (100%) patients in the orphenadrine group reported dry mouth.
Tizanidine	Fogelholm<br>Finland<br>1992	Crossover RCT	Women with chronic tension-type headaches randomized to tizanidine vs placebo in six-week blocks	37	- Median visual analog scale significantly improved in tizanidine group vs. placebo group.<br>- Median verbal rating scale significantly improved in tizanidine group vs. placebo group.<br>- Tizanidine group experienced dry mouth and drowsiness and the placebo group did not. Two patients dropped out due to these adverse effects.
	Krusz<br>USA<br>2000	Cohort	Patients with chronic tension-type headaches received tizanidine for three months	222	- Patients reported a mean 72% reduction in frequency of headaches; severity reduced by 62%, and an improvement in sleep in 95%.<br>- Among the 222 patients, 55 (25%) dropped out, among them 16 (7%) due to adverse events, including vivid dreams/nightmares, hallucinations, and dry mouth.
	Murros<br>Finland<br>2000	RCT	Patients with chronic tension-type headaches randomized to tizanidine vs. placebo for six weeks	105<br>(55 received placebo)	- Visual analog pain scale significantly improved in placebo and two dose groups of tizanidine; no significant differences noted between groups.<br>- Mean duration of headaches improved in all groups; no significant differences noted between groups.<br>- Tiredness (17%) and dry mouth (22%) most common adverse events in the tizanidine group.
	Shimomura<br>Japan<br>1991	Cohort	Patients with chronic tension-type headaches	78	- 24 (31%) reported “excellent improvement,” 28 (35%) reported “moderate improvement”, 18(23%) reported “mild improvement,” and 7 (10%) reported no improvement after four weeks.

© 2024 Oldfield BJ et al. *JAMA Network Open.*

			given tizanidine for four weeks		- Some (not quantified) patients reported drowsiness, epigastric discomfort, but none discontinued the drug
Baclofen	Parmar India 1989	Cohort	Patients with chronic trigeminal neuralgia given baclofen for two months	20	- Nine (45%) patients reported complete resolution of symptoms; 4 (20%) exhibit partial resolution.<br>- Seven (35%) exhibited no response and opted for standard treatment.<br>- Seven (35%) patients reported adverse effects: sedation, vomiting, diarrhea, nausea.
	Puri India 2018	RCT	Patients with chronic trigeminal neuralgia randomized to carbamazepine, carbamazepine with baclofen, and carbamazepine with capsaicin for one month	17 (16 received carbamazepine alone, 16 received carbamazepine with capsaicin)	- All groups showed significant reduction in mean visual analog pain scales.<br>- Comparative reduction in pain was significantly better in carbamazepine with baclofen group compared to carbamazepine alone group at 15 days and 1 month.<br>- Adverse effects were noted in three (18%) patients in carbamazepine with baclofen group: nausea, drowsiness, weakness, constipation.
	Steardo Italy 1984	Cohort	Patients with chronic trigeminal neuralgia (16) and other chronic painful conditions (9) given baclofen for six months	25	- Among those with trigeminal neuralgia, the "amelioration rate" was 71% in those previously untreated; 60% in those previously trialed on cyclobenzaprine.<br>- Adverse effects not commented upon.
	Kookna India 2022	RCT	Patients with chronic trigeminal neuralgia randomized to carbamazepine, carbamazepine plus baclofen, and carbamazepine	20 (20 received carbamazepine alone, 20 received carbamazepine plus capsaicin	- All groups reported improvement in pain control.<br>- The baclofen group's average report of pain was significantly less at 30 days than the two comparator groups.<br>- Adverse effects were not commented upon.

© 2024 Oldfield BJ et al. *JAMA Network Open.*

			plus capsaicin for 30 days		
Cyclobenzaprine	Mueller<br>USA<br>2014	RCT	Patients with chronic migraine randomized to extended-release cyclobenzaprine vs. placebo for 12 weeks	15<br>(16 received placebo)	- Compared to placebo, cyclobenzaprine did not result in significant reduction in mean migraine headache days.

© 2024 Oldfield BJ et al. *JAMA Network Open.*

eTable 4. Characteristics of Interventions for Painful Muscle Cramps or Spasticity

Medication	First author, country, publication year	Study design	Intervention description	Number of patients receiving intervention	Key findings
Methocarbamol	Abd-Elsalam Egypt 2018	RCT	Patients with cirrhosis and frequent cramps randomized to methocarbamol vs. placebo for one month	50 (50 received placebo)	- Mean number of cramps/week significantly lower in methocarbamol (0.5) versus placebo (9)<br>- Mean duration of cramps significantly lower in methocarbamol (0.71 min) versus placebo (3.9 min)<br>- Side-effects in methocarbamol included drowsiness (24%) and dry mouth (18%)<br>- Two weeks after stoppage of treatment, no clinically meaningful differences in groups
Orphenadrine	Abd-Elsalam Egypt 2019	RCT	Patients with cirrhosis and frequent cramps randomized to ophenadrine vs. placebo for one month	62 (62 received placebo)	- Mean number of cramps/week significantly lower in orphenadrine (0.91) versus placebo (11.9)<br>- Mean duration of cramps significantly lower in orphenadrine (1.86 min) versus placebo (3.90 min)<br>- No difference in side-effects between groups<br>- Two weeks after stoppage of treatment, frequency (1.90 versus 10.6) and duration (1.90 versus 4.67 min) significantly more favorable in orphenadrine group
	Popkin USA 1971	Cohort	Patients with painful nocturnal leg cramps given orphenadrine for periods from three months to one year	32	- Five (16%) patients benefited within one month and did not require further medication.<br>- Eleven (34%) patients benefited as long as orphenadrine was continued (one year).<br>- Twelve (38%) dropped out due to gastrointestinal intolerance within the first four days.
Baclofen	Aydin Turkey 2005	RCT	Patients with traumatic spinal-cord injury and painful spasticity randomized to enteral baclofen vs. transcutaneous electrical nerve stimulation	10 (11 received TENS)	- Significant improvement (before versus at eight weeks) was detected in lower limb Ashworth score, spasm frequency score, deep-tendon reflect score and functional disability score, in both the baclofen and TENS groups.<br>- No differences were detected in comparisons between baclofen and TENS groups.<br>- Side-effects not quantified.

© 2024 Oldfield BJ et al. *JAMA Network Open.*

			(TENS) for eight weeks		
	Elfert<br>Egypt<br>2016	RCT	Patients with cirrhosis and frequent cramps randomized to baclofen versus placebo for 3 months	50<br>(50 received placebo)	- Significant improvement in frequency, duration, and severity of cramps noted in baclofen group. No improvement noted in placebo group.<br>- No significant difference in quality of life in baclofen vs. placebo group.<br>- No significant difference in adverse effects between the two groups.
	Henry<br>USA<br>2014	Cohort	Patients with cirrhosis and frequent cramps given baclofen for five weeks	10	- Significant improvement in mean muscle cramp frequency (5.5 days/week to 1.4) and severity (8.5/10 to 2.8/10).<br>- One (10%) patient discontinued baclofen due to somnolence, another decreased dose due to somnolence.
Carisoprodol	Chesrow<br>USA<br>1963	RCT	Patients with leg cramps associated with vascular, neurologic, or arthritic disease randomized to carisoprodol vs. placebo for 10 months	26<br>(19 received placebo)	- In the carisoprodol group, 21 (81%) reported "definite improvement in leg pain, muscle spasm, and tenderness."<br>- In the placebo group, 4 (21%) reported slight relief in cramps.<br>- No measures of association were reported.<br>- Side effects of carisoprodol were reported to be "mild and transient and did not necessitate discontinuance of the medication."
	Stern<br>USA<br>1963	Crossover RCT	Inpatient nursing home patients with chronic, painful leg cramps randomized to carisoprodol vs. placebo for four weeks	50	- In 41 (82%) patients, response reported as "excellent" or "good" to carisoprodol, whereas only 1 (2%) reported "excellent" or "good" response to placebo.<br>- Drowsiness was noted in 17 (32%) of patients receiving carisoprodol; none discontinued carisoprodol.
Quinine	Connolly<br>USA<br>1992	Crossover RCT	Veterans with nocturnal leg cramps randomized to quinine sulfate, vitamin E, or	27	- When receiving quinine, patients reported significantly fewer cramps per month (19) than placebo (37) or vitamin E (32), but the severity score of the cramps were not significantly different.<br>- Total side effects were significantly greater in the quinine groups than vitamin E or placebo.

© 2024 Oldfield BJ et al. *JAMA Network Open.*

			placebo for four week blocks		
	Woodfeld<br>New Zealand<br>2005	Crossover RCT	Patients with chronic painful leg cramps randomized to quinine vs. placebo for four week blocks	13	- Ten individuals completed the trial. Total number of cramps improved during quinine block for three (30%) participants, and seven (70%) did not show significant improvement in number of cramps.<br>- No differences in adverse effects noted during quinine blocks.

eTable 5. Characteristics of Interventions for Other Pain Syndromes

Medication	First author, country, publication year	Study design	Intervention description	Number of patients in intervention	Key findings
Baclofen	Mohseni-Rad<br>Iran<br>2020	Cohort	Patients with chronic orchialgia given baclofen or terazosin for three months	244<br>(255 received terazosin)	- Chronic prostatitis symptom scores improved significantly for both baclofen (25/43 to 20/43) and terazosin groups (25/43 to 20/43).<br>- No significant differences observed between the baclofen and terazosin groups.<br>- No complications were reported in either group.
	Yomiya<br>Japan<br>2009	Cohort	Retrospective chart review of with cancer pain given baclofen for average of 230 days	25	- Of 25 patients, 21 (84%) had 50% or greater pain reduction.<br>- Sleepiness appeared in 6 (24%) patients.
	Pauwels<br>Belgium<br>2022	RCT	Patients with refractory gastric reflux symptoms randomized to baclofen versus placebo for four weeks	31<br>(28 received placebo)	- General wellbeing and reflux-related complaints improved significantly in both groups.<br>- In subgroup analyses, those with symptom-association probability (based on pH impedance monitoring) improved significantly with baclofen and worsened with placebo.<br>- Five patients dropped out of the study, all in the baclofen group (16%) due to drowsiness, dizziness, headache and/or nausea.
Chlormezanone	Berry<br>UK<br>1980	Crossover RCT	Patients with degenerative disease of hip, knee, spine, or shoulder randomized to benorylate with or without chlormezanone for four weeks	90	- Chlormezanone significantly reduced number of breaks in sleep and improved pain relief in those with neck pain.<br>- No significant difference noted in patients with degenerative disease of hip, knee, lumbar spine, or shoulder.<br>- Drowsiness more common in chlormezanone groups.
Cyclobenzaprine	Elchami<br>Saudi Arabia<br>2011	RCT	Patients with neuropathic pain associated with	160	- Mean improvement in numeric pain scale 75% in pregabalin plus cyclobenzaprine group versus 60% in pregabalin-alone group.

© 2024 Oldfield BJ et al. *JAMA Network Open.*

			radiculopathy randomized to pregabalin with or without cyclobenzaprine for three months	(140 received pregabalin alone)	- Benefit sustained for three months in both groups.<br>- No measures of association documented.<br>- No side effects documented.
Eperisone	Kaur<br>India<br>2013	RCT	Patients with knee osteoarthritis randomized to etodolac with or without eperisone for eight weeks	30<br>(30 received etodolac alone)	- Significant improvement in both groups in mean scores of spontaneous pain on visual analog scale, pain on movement, functional capacity, joint tenderness, swelling, erythema, and overall condition.<br>- No significant differences observed between groups.<br>- Adverse events few and mild.
	Bose<br>Singapore<br>1999	RCT	Patients with cervical spondylosis randomized to eperisone vs. placebo for six weeks.	75<br>(82 received placebo)	- Both groups showed improvement in pain severity and range of motion.<br>- Improvement in neck pain significantly improved in eperisone group over placebo group at week six.<br>- Adverse effects occurred in 4.2% of those in eperisone group; these adverse effects were not classified further.
Tizanidine	Semenchuk<br>USA<br>2000	Cohort	Patients seeking care for neuropathic pain at a neurology clinic given tizanidine for eight weeks	23	- Mean average weekly pain score decreased by 1.7 points at end of week 8 (from 6.9 to 5.2, P <0.01).<br>- 15 patients (68%) reported pain relief improved or much-improved.<br>- Adverse effects included dizziness (52%), drowsiness (48%), fatigue (43%), dry mouth (39%). One patient discontinued due to hepatic transaminase elevation.

© 2024 Oldfield BJ et al. *JAMA Network Open.*

eTable 6. Risk of Bias Assessment for Randomized Trials (Cochrane Risk of Bias Tool)

Study	Random sequence generation	Allocation concealment	Blinding of participants and personnel	Blinding of outcome assessment	Incomplete outcome data	Selective Reporting	Other sources of bias
Abd-Elsalam 2017	Low risk	Low risk	Low risk	Low risk	Low risk	Low risk	Low risk
Abd-Elsalam 2018	Low risk	Low risk	Low risk	Low risk	Low risk	Low risk	Low risk
Aydin 2005	High risk	High risk	High risk	Low risk	Low risk	Low risk	Low risk
Bakris 1982	Low risk	Low risk	High risk	Unclear risk	Low risk	Low risk	High risk
Bennett 1988	Low risk	Unclear risk	Unclear risk	Low risk	High risk	Low risk	Low risk
Berry 1980	Low risk	Low risk	Low risk	Low risk	High risk	Low risk	Low risk
Bose 1999	Low risk	Low risk	Low risk	Low risk	High risk	Low risk	High risk
Cantini 1994	Low risk	Low risk	Unclear risk	Unclear risk	Low risk	Low risk	Low risk
Carette 1994	Low risk	Low risk	Low risk	Unclear risk	Low risk	Low risk	Low risk
Chesrow 1963	Low risk	Low risk	High risk	Low risk	Low risk	Low risk	High risk
Connolly 1992	Low risk	Low risk	Low risk	Low risk	High risk	Low risk	Low risk
Elchami 2011	Unclear risk	Unclear risk	Unclear risk	High risk	Unclear risk	Unclear risk	Low risk
Elfert 2016	Low risk	Low risk	Low risk	Low risk	Low risk	Low risk	Low risk
Fogelholm 1992	Low risk	Low risk	Unclear risk	Unclear risk	Low risk	Low risk	Low risk
Kaur 2013	Low risk	Low risk	Unclear risk	Unclear risk	Low risk	Low risk	Low risk
Kookna 2022	Unclear risk	High risk	High risk	High risk	High risk	Low risk	High risk
Lederman 2015	Unclear risk	Unclear risk	Low risk	Low risk	Unclear risk	Unclear risk	Low risk
Mueller 2014	Unclear risk	Unclear risk	Unclear risk	Unclear risk	Low risk	Low risk	Low risk
Murros 2000	Low risk	Low risk	Low risk	Low risk	High risk	High risk	Low risk
Pattrick 1993	Unclear risk	Low risk	Low risk	Low risk	Low risk	Low risk	Low risk
Pauwels 2022	Unclear risk	Low risk	Low risk	Low risk	High risk	Low risk	Low risk
Puri 2018	Unclear risk	High risk	High risk	High risk	Low risk	Low risk	Low risk
Quimby 1989	Unclear risk	Unclear risk	High risk	High risk	Low risk	Low risk	Low risk
Rossi 2012	Unclear risk	High risk	High risk	Low risk	Low risk	Low risk	Low risk
Sakai 2008	Unclear risk	Low risk	High risk	High risk	Low risk	Low risk	High risk
Stern 1963	Unclear risk	Low risk	Low risk	Low risk	Low risk	Low risk	Low risk
Vaerøy 1989	Unclear risk	Low risk	Low risk	Low risk	High risk	Low risk	Low risk
Williamson 2000	Low risk	Low risk	Low risk	Low risk	Low risk	Low risk	Low risk
Woodfield 2005	Low risk	Low risk	Low risk	Low risk	High risk	Low risk	High risk

© 2024 Oldfield BJ et al. *JAMA Network Open.*

Zaringhalam 2010	Unclear risk	High risk	High risk	High risk	Low risk	Low risk	Low risk

© 2024 Oldfield BJ et al. *JAMA Network Open.*

eTable 7. Quality Assessment for Cohort Studies (Newcastle-Ottawa Scale)

Study	Representative exposed cohort	Selection of the non-exposed cohort	Ascertainment of exposure	Demonstration that outcome of interest was not present at start of study	Comparability of cohorts on the basis of the design or analysis	Assessment of outcome	Follow-up long enough for outcomes to occur	Adequacy of follow up of cohorts
Henry 2014	*			*		*	*	
Krusz 2000	*		*	*		*	*	*
Malanga 2002	*		*	*		*	*	*
McClain 2002	*		*	*		*	*	*
Mohseni-Rad 2020	*	*	*	*		*	*	
Parmar 1989	*		*			*	*	*
Popkin 1971				*		*	*	
Reale 2012	*			*			*	
Semenchuck 2000	*		*	*		*	*	
Shimomura 1991			*	*			*	
Steardo 1984							*	
Ueberall 2022	*	*	*	*	*	*		
Xiao 2002	*		*	*			*	
Yomiya 2009				*			*	

© 2024 Oldfield BJ et al. *JAMA Network Open.*
