# Supplementary material for: Long-Term Use of Muscle Relaxant Medications for Chronic Pain: A Systematic Review
Source: JAMA Netw Open. 2024 Sep 19;7(9):e2434835. doi: 10.1001/jamanetworkopen.2024.34835 (PMC11413720; doi:10.1001/jamanetworkopen.2024.34835)
Supplement: Supplement 2. — Data Sharing Statement [file jamanetwopen-e2434835-s002.pdf]

Data Sharing Statement

Oldfield. Long-Term Use of Muscle Relaxant Medications for Chronic Pain. *JAMA Netw Open*. Published September 19, 2024. doi:10.1001/jamanetworkopen.2024.34835

Data
Data available: No
